# Implementation and adoption of a health insurance support tool in the electronic health record: a mixed methods analysis within a randomized trial

**DOI:** 10.1186/s12913-020-05317-z

**Published:** 2020-05-15

**Authors:** Brigit Hatch, Carrie Tillotson, Nathalie Huguet, Miguel Marino, Andrea Baron, Joan Nelson, Aleksandra Sumic, Deborah Cohen, Jennifer E. DeVoe

**Affiliations:** 1 Oregon Health & Science University, 3405 SW Perimeter Court, Portland, OR 97239 USA; 2 OCHIN, 1881 SW Naito Parkway, Portland, OR 97201 USA

**Keywords:** Community health centers, Health insurance, Outreach and enrollment, Health information technology tools, Electronic health record, Hybrid implementation-effectiveness, Mixed methods

## Abstract

**Background:**

In addition to delivering vital health care to millions of patients in the United States, community health centers (CHCs) provide needed health insurance outreach and enrollment support to their communities. We developed a health insurance enrollment tracking tool integrated within the electronic health record (EHR) and conducted a hybrid implementation-effectiveness trial in a CHC-based research network to assess tool adoption using two implementation strategies.

**Methods:**

CHCs were recruited from the OCHIN practice-based research network. Seven health center systems (23 CHC clinic sites) were recruited and randomized to receive basic educational materials alone (Arm 1), or these materials plus facilitation (Arm 2) during the 18-month study period, September 2016–April 2018. Facilitation consisted of monthly contacts with clinic staff and utilized audit and feedback and guided improvement cycles. We measured total and monthly tool utilization from the EHR. We conducted structured interviews of CHC staff to assess factors associated with tool utilization. Qualitative data were analyzed using an immersion-crystallization approach with barriers and facilitators identified using the Consolidated Framework for Implementation Research.

**Results:**

The majority of CHCs in both study arms adopted the enrollment tool. The rate of tool utilization was, on average, higher in Arm 2 compared to Arm 1 (20.0% versus 4.7%, *p < 0.01).* However, by the end of the study period, the rate of tool utilization was similar in both arms; and observed between-arm differences in tool utilization were largely driven by a single, large health center in Arm 2. Perceived relative advantage of the tool was the key factor identified by clinic staff as driving tool utilization. Implementation climate and leadership engagement were also associated with tool utilization.

**Conclusions:**

Using basic education materials and low-intensity facilitation, CHCs quickly adopted an EHR-based tool to support critical outreach and enrollment activities aimed at improving access to health insurance in their communities. Though facilitation carried some benefit, a CHC’s perceived relative advantage of the tool was the primary driver of decisions to implement the tool.

**Trial registration:**

ClinicalTrials.gov: NCT02355262, Posted February 4, 2015.

## Background

Community Health Centers (CHCs) provide a vital source of health care to more than 28 million people in the United States. Because CHCs accept patients regardless of their ability to pay, these health centers care for a large proportion of patients with no health insurance, patients with frequent health insurance coverage gaps, and patients with Medicaid insurance [1]. The Patient Protection and Affordable Care Act (ACA) greatly expanded access to health insurance and also increased funding to CHCs via the Community Health Center Fund [2].

Because of the increased access to health insurance coverage and the insurance enrollment complexity following the ACA, the Health Resources and Services Administration (HRSA) provided grant-funding to over 1000 CHCs to support health insurance outreach and enrollment (O&E) efforts [3]. This grant funding supported many CHCs in establishing health insurance enrollment assisters to help patients enroll and re-enroll in health insurance, especially Medicaid. Patients who require assistance with insurance may self-identify for these services, may be referred at the time of an appointment, or may be identified for outreach outside of the context of an appointment [4]. Individual CHCs develop their own systems for O&E supports, thus models for providing these services vary widely, as do the systems for tracking outreach.

To support health insurance enrollment assisters in their work to improve health insurance enrollment and continuity (a widely demonstrated benefit to patient health) {DeVoe, 2003 #193} {Hatch, 2017 #492}, our team developed and implemented an electronic tool integrated within the electronic health record (EHR) – referred to as the ‘enrollment tool.’ The enrollment tool was designed to streamline and improve tracking of O&E services, with the ultimate goals of increasing insurance continuity, reducing uninsured visits, and improving patient care [5]. We used a mixed method, hybrid effectiveness-implementation design [6, 7] to study adoption of the enrollment tool. As health centers are increasingly asked to implement new strategies to streamline and improve care, just as this tool was conceptualized to do, it is critical to understand which implementation strategies best support practice change (or not) and why. In this manuscript, we report on the implementation component of this study’s hybrid effectiveness-implementation design and compare tool utilization outcomes among two implementation strategies: (1) basic educational materials (Arm 1); and (2) basic educational materials plus facilitation (Arm 2) — a strategy that evidence shows can assist practices with implementing change, such as adopting evidence-based guidelines [8, 9].

## Methods

### Study setting & participants

Primary care health centers were recruited from the OCHIN (not an acronym) practice-based research network (PBRN) [10]. OCHIN is the largest network of CHCs using a single instance of the Epic EHR. Its centrally hosted EHR is deployed in over 100 health center systems (595 clinic sites) caring for nearly 3.6 million patients across 22 states. For this study, eligible OCHIN health centers met the following criteria: located in a state that expanded Medicaid in 2014, implemented the OCHIN EHR prior to 2013, and no history of participation in a study of similar tools targeted toward children [11]. Of 32 eligible health centers, 10 were not approached because they were being recruited for other OCHIN PBRN projects. Of the 22 health centers invited, seven (31.8%) agreed to participate. This study adhered to CONSORT guidelines (Appendix Fig. 1). The seven participating health centers were composed of 23 individual clinic sites (we will refer to the larger entities as health centers, recognizing that most health centers are systems with more than one affiliated clinic site). The health centers included in this study ranged from one to six clinics per health center, and all were designated Federally Qualified Health Centers (FQHCs). All participating clinics sites received HRSA grant funding for O&E. Quantitative data were available for all 7 health centers throughout the entire study period, however, one health center in Arm 1 (Health Center D) was lost to qualitative follow up.

### Study design and intervention

This project was designed as a hybrid effectiveness-implementation study to identify the factors that explain why some health centers implemented the tools and others did not. More detailed description of study design can be found in the study protocol by DeVoe [5]. The tool implementation period consisted of 18 months – September, 2016 through March, 2018. This period was preceded by a 6-month tool testing and refinement window (March–September, 2016) where a preliminary version of the tool was released to all participating clinics and Arm 2 clinics were engaged in ‘beta testing.’ The final version of the tool was released to all clinics in Arms 1 and 2 mid-September, 2016. Qualitative data collection continued through July, 2018. The intervention included the enrollment tool, educational materials, ‘beta testing,’ and facilitation which are described below [12].

#### The enrollment tool

As described in Table 1, the enrollment tool consisted of an electronic fillable ‘form’ which appeared alongside typical patient registration processes within the EHR. This form was intended for use by enrollment assisters or other staff who help patients or community members with registration or insurance enrollment. Each ‘form’ can be used to assist multiple individuals and is electronically linked to a single individual’s health record. Screenshot of the Enrollment Tool is included in the appendix (Appendix Fig. 2).

**Table 1:** Enrollment tool functionality

➢ *Tracking and Documenting*: Fillable fields to collect and document insurance enrollment information such as status of insurance application, insurance ID, effective date, eligibility status, number and type of assists provided, total number of individuals assisted, notes, etc.	
➢ *Panel management function*: Allows users to (1) run a report of patients with upcoming appointments within 30 days and identify those without health insurance; and (2) run a daily report of health insurance application assistance in progress.	
➢ *Retrospective data report*: Reports the number of total individuals assisted with each opened form to generate HRSA quarterly reporting of outreach and enrollment assistance provided.	

#### Educational materials

All participating health centers received basic educational materials consisting of an electronic manual with instructions for tool use.

#### ‘Beta testing’

Only Arm 2 clinics participated in an initial period of beta testing (the 6 months prior to the study period) which included working with the facilitator and participating in user-centered design feedback sessions that led to tool refinement and updates.

#### Facilitation

In addition to basic educational materials, Arm 2 clinics received facilitation customized to meet individual practice needs. During the 18-month implementation study period, the facilitator contacted all Arm 2 health centers (by phone, virtual meeting space, or email) at least monthly. During these contacts, the facilitator provided audit and feedback reports which included graphs of monthly rates of uninsured and Medicaid visits, as well as monthly use of the enrollment tool, for each of the clinic sites and the composite health center. The facilitator also provided tailored guidance to support clinic-led rapid change cycles focusing on enhancing tool utilization. Throughout the study period, the facilitator was also able to expedite problem-solving (including technologic support) for any questions/concerns regarding study tools for Arm 2 clinics.

### Randomization procedure

The study team randomized four health centers (11 clinic sites) to Arm 1 which received educational materials only, and three health centers (12 clinic sites) to Arm 2, which received educational materials plus facilitation. Participating clinic sites were randomized by health center system into one of two intervention arms through covariate-constrained randomization. We used state (Oregon vs. non-Oregon), number of clinics per health center, total number of patients per health center, and percentage of uninsured patients per health center as covariates in our random procedure as these were theorized to be important confounders. Given the focus on comparing intervention arms on tool adoption, we do not report on how we selected controls for testing effectiveness as these are beyond the scope of this paper and can be found in the study protocol paper [5].

### Quantitative data

The primary quantitative outcome of interest was tool use during the study period. First, we measured individual instances of tool use and number of unique patients for whom the tool was used. Then, because health center size and population varied widely, we quantified tool use as a rate. We set the numerator as the number of unique patients with tool use who had ≥1 Medicaid-insured or uninsured clinical visit at an intervention CHC during the study period, and the denominator as the total number of CHC patients with ≥1 Medicaid-insured or uninsured clinical visits at an intervention CHC during the study period (presumed to be individuals at highest risk of insurance discontinuity). An instance of tool use was defined as any instance where the form was opened and saved.

For descriptive purposes, we collected patient- and CHC-level information rolled up to the health center. Patient-level characteristics included patient status at time of first tool use (established patient, new patient, or never patient); and insurance type prior to first tool use (Medicaid, Medicare, private, other public, Uninsured, no prior visits/missing). CHC characteristics included number of active patients (individuals with an ambulatory encounter during the study period); % uninsured ambulatory visits during the implementation period; % Medicaid-insured ambulatory visits during the implementation period; % nonwhite; % Hispanic; median patient age (years); % Federal poverty level (FPL) < 138% at the beginning of the implementation period; number of clinics within their health center system; and urbanicity (rural, urban, mixed—one health center had clinic sites in both rural and urban locations).

### Qualitative data

Qualitative data collection included ethnographic observation and semi-structured interviews with key stakeholders at CHCs and was focused on understanding why (or why not) and how the enrollment tool was implemented, with attention to differences between each arm. Data were collected through in-person site visits to CHC clinics, and through monthly phone interviews with CHC contacts. Interview guide and field definitions are included in the appendix (Appendix Tables 1 and 2).

Two experienced researchers conducted at least one in-person site visit with all but one of the CHCs – Health Center D did not participate in a site visit. Site visits focused on identifying practices’ experiences with the enrollment tool, including how the tool was integrated into clinical work flows and the barriers and facilitators to tool use. Health centers in Arm 2 additionally received a baseline site visit aimed at assessing existing clinic O&E processes prior to intervention, understanding motivation for using the enrollment tool, and understanding aspects of practice organizational capacity, including existing tools used for O&E. Health centers generally participated in site visits according to their own willingness, capacity, and clinic structure (one at A, C, and E; two at B and F, and three at G). Site visits lasted 1–2 days and included 8–14 h of observation, 3–6 semi-structured interviews with practice staff (*n* = 47) utilizing snowball recruitment technique to identify all staff who were directly or indirectly involved in tool use (e.g.*,* front desk staff, leadership, outreach and enrollment staff, heath information technology support), and the collection of artifacts related to the enrollment process (e.g.*,* enrollment applications, training and educational materials). The number of interviews conducted at each clinic varied by clinic size and captured all available clinic-identified stakeholders in the health insurance outreach and enrollment process. Interviews were approximately 45–60 min long.

In addition to site visit data, one researcher made monthly phone contact with each CHC during the study period, targeting one to two key informants who were most closely involved with implementation of the enrollment tool. Phone interviews were approximately 20 min long and included discussion of tool implementation, enrollment patterns, and experience with implementation support.

### Quantitative analysis

First, we described patient and clinic characteristics by health center and study arm. Similarly, we compared several characteristics of tool utilization by health center and study arm. We compared rates of tool use between Arms 1 and 2 using a two-sided Poisson exact rate ratio test, and report the rate ratio (95% confidence interval) comparing Arm 2 to Arm 1. Statistical significance was set at α < 0.05. To maximize learnings from individual health centers, we also report descriptive rates of tool use by health center. Lastly, to understand uptake and continued tool use throughout the implementation period, we visually present trends in tool utilization over time by estimating monthly rates of tool use by study arm and for each health center. Quantitative analyses were performed using SAS software, v.9.4 (SAS Institute Inc., Cary, NC) and R version 3.6.0 [13].

### Qualitative data management and analysis

Interviews were audio-recorded, professionally transcribed, and checked for errors. Jottings made in the field were developed into comprehensive field notes within 24–48 h of the site visit’s end. Data collection and analyses were iterative with initial findings guiding subsequent interview questions [14]. Fieldnotes and interview transcripts were de-identified and put into Atlas.ti (Version 7.0, Atlas.ti Scientific Software Development GmbH, Berlin, Germany) for data management and analysis.

We used an immersion/crystallization approach [15] to analyze the data. Two experienced qualitative analysts read the data from each health center (immersion) and then regularly met to discuss patterns within each site (crystallization). Through this process, the team developed a codebook to tag relevant portions of the text. Data were then analyzed a second time to draw comparisons across health centers. This process yielded patterns in the factors that influenced use of the enrollment tool, and we began to make connections to relevant literature to help enhance and explain emerging results. This methodology has been used elsewhere in qualitative primary care research [16–18].

We used the Consolidated Framework for Implementation Research (CFIR) [19] to help name the barriers and facilitators to implementation that we observed. Lastly, we developed a matrix organized by health center and study arm, and input qualitative data for each of the factors identified as influencing tool use. This allowed the team to organize cross-cutting findings and to develop a more robust understanding of what happened among participating health centers and why [20].

### Quantitative/qualitative data integration

Throughout the study period, quantitative and qualitative analytic teams worked in parallel to analyze the data while minimizing bias. When analyses were complete, the results were examined by the full study team to identify common themes explaining the observed results and to integrate the presentation of data. With assistance of the full study team, qualitative and quantitative results were triangulated and contextualized within the broader literature and study design. This methodology has been used elsewhere and is noted to maximize rigor in mixed methods analyses [21].

This study was approved by the Institutional Review Board at Oregon Health & Science University and was registered as an observational study at clinicaltrials.gov (NCT02355262).

## Results

The seven health centers (with 23 study clinics) had patient populations ranging in size from 3584 patients to 22,286 patients during the implementation period (Table 2). The rate of uninsured visits varied across clinics, from 13.9 to 52.8%. In all but one health center, more than 40% of encounters were Medicaid-insured. Characteristics of the patient population that health centers served (e.g., race and ethnicity) varied.

**Table 2:** Characteristics of participating health centers

	**ARM 1 (basic education material)**				**ARM 2 (basic education materials + facilitation)**		
	**Health Center A**	**Health Center B**	**Health Center C**	**Health Center D**	**Health Center E**	**Health Center F**	**Health Center G**
Number active patients^a^	3584	20,830	4368	4334	13,301	4020	22,286
Number clinics	3	4	3	1	5	1	6
% Uninsured visits	17.5	20.1	20.2	52.8	13.9	19.8	17.8
% Medicaid insured visits	42.7	52.3	47.1	41.5	61.7	26.4	51.8
Urbanicity^b^	Urban	Urban	Mixed	Urban	Urban	Rural	Urban
% Nonwhite	4.8	24.1	2.4	11.0	21.0	1.0	2.8
% Hispanic	6.5	26.3	26.9	49.3	61.4	2.5	18.1
Median age (years)	52.6	33.2	37.7	43.5	46.8	53.1	46.6
% Income < 138% FPL^c^	70.1	78.4	39.1	85.0	53.2	31.6	37.5

a.) Active patients defined as individuals with a ambulatory visit during the study period (September, 2016-March, 2018)

b.) Urbanicity defined as all clinic sites located in urban areas (≥2500 residents), all clinics located in rural areas (< 2500 residents), or mixed (clinics located in both urban and rural areas). Urban and rural areas determined according to the 2010 US Census.

c.) *FPL* Federal Poverty Level

### Most clinics utilized the tool

From Table 3, five of seven health centers recorded tool use. One health center in Arm 1 (D) and one health center in Arm 2 (E) did not record any tool use. Health Center G (Arm 2) had the highest rate of tool utilization, with over five times more unique patients with tool use and just over 4 times more instances of tool use compared to the next closest health center. The population for whom the tool was used was generally similar across health centers, and most were uninsured or Medicaid-insured patients. However, at Health Center A, 44% of individuals who received insurance support with the enrollment tool never became patients at that health center during the study period, demonstrating that their health center utilized the tool to engage in more community enrollment support than other CHCs. Though most tool utilization assisted one person per encounter, many unique instances of tool use assisted more than one person. At health center C, 49% of tool use instances assisted multiple individuals while at health center G, only 22% of tool use instances assisted multiple people.

**Table 3:** Tool use by health center during the implementation period, September, 2016-March, 2018

	**ARM 1**				**ARM 2**		
	**Health Center A**	**Health Center B**	**Health Center C**	**Health Center D**	**Health Center E**	**Health Center F**	**Health Center G**
Unique patients with tool use	662	279	1600	0	0	432	8403
Total instances^a^ of tool use	747	374	3047	0	0	609	13,068
Patient Status							
Established patient	329 (49.7)	220 (78.9)	1418 (88.6)	0 (0.0)	0 (0.0)	402 (93.1)	7382 (87.9)
New patient	41 (6.2)	28 (10.0)	50 (3.1)	0 (0.0)	0 (0.0)	11 (2.6)	243 (2.9)
Never patient	292 (44.1)	31 (11.1)	132 (8.2)	0 (0.0)	0 (0.0)	19 (4.4)	778 (9.3)
Total # persons assisted							
No Information	1 (0.2)	2 (0.7)	66 (4.1)	0 (0.0)	0 (0.0)	12 (2.8)	146 (1.7)
1	452 (68.3)	319 (78.5)	750 (46.9)	0 (0.0)	0 (0.0)	275 (63.7)	6399 (76.2)
> 1	209 (31.6)	58 (20.8)	784 (49.0)	0 (0.0)	0 (0.0)	145 (33.6)	1858 (22.1)
Insurance type prior to first tool use							
Medicaid	183 (27.6)	46 (16.5)	779 (48.7)	0 (0.0)	0 (0.0)	296 (68.5)	5543 (66.0)
Medicare	15 (2.3)	4 (1.4)	31 (1.9)	0 (0.0)	0 (0.0)	7 (1.6)	85 (1.0)
Private	28 (4.2)	19 (6.8)	240 (15.0)	0 (0.0)	0 (0.0)	51 (11.8)	352 (4.2)
Other public^b^	16 (2.4)	1 (0.4)	40 (2.5)	0 (0.0)	0 (0.0)	0 (0.0)	72 (0.9)
Uninsured	64 (9.7)	114 (40.9)	315 (19.7)	0 (0.0)	0 (0.0)	37 (8.6)	1171(14.0)
No prior visits/Missing	356 (53.8)	95 (34.1)	195 (12.2)	0 (0.0)	0 (0.0)	41 (9.5)	1180 (14.0)
Number of staff using the tool	3	5	8	0	0	5	10

a.) Instances of tool use defined as unique patient-dates of tool use

b.) Other Public Insurance includes publically-funded coverage sources typically covering limited services (e.g., breast and cervical cancer early detection program; title X contraceptive care) or available to specific populations (e.g., VA and Tricare, Indian Health Service, grant programs for migrant/seasonal workers, and care for the homeless or individuals living with HIV/AIDS)

### For patients at high risk of uninsurance, arm 2 health centers used the tool more frequently than arm 1 health centers

Among the population we defined as at highest risk of insurance discontinuity – those with at least one Medicaid-insured or uninsured ambulatory visit during the study period (*n* = 51,656) – a total of 6602 (12.8%) unique patients received insurance support with the enrollment tool (Table 4). Overall, the rate of patients with at least one Medicaid-insured or uninsured visit who received support with the enrollment tool was significantly higher in Arm 2 health centers compared to those in Arm 1 (20.0% vs 4.7%, *p < 0.01*, RR = 4.27 95%, CI = 4.01–4.56). Table 4 also demonstrates variability in rate of tool use between health centers with a higher rate of tool use by Health Center G (33%) and a much lower rate of tool use at health center B (1.5%).

**Table 4:** Comparison of tool utilization (by health center and study arm) among patients with ≥1 Medicaid-covered or uninsured ambulatory visit during the implementation period (September, 2016-March, 2018)

	**Arm 1**				**Arm 2**			**RR**^**a**^**(95% CI)**
	**Health Center A**	**Health Center B**	**Health Center C**	**Health Center D**	**Health Center E**	**Health Center F**	**Health Center G**	
Number patients with tool use	211	226	696	0	0	347	5122	
Total number of patients	2155	15,066	2942	4087	10,049	1856	15,501	
Percent of patients with tool use (by CHC)	9.8%	1.5%	23.7%	0.0%	0.0%	18.7%	33.0%	
Percent of patients with tool use (by arm)	4.7%				20%			4.27 (4.01, 4.56) *p* < 0.001

a.) *RR* Rate Ratio comparing study arms, *CI* Confidence Interval

### Tool utilization varied over time, with similar tool utilization rates in study arms 1 and 2 by the end of the study

**Fig. 1:**
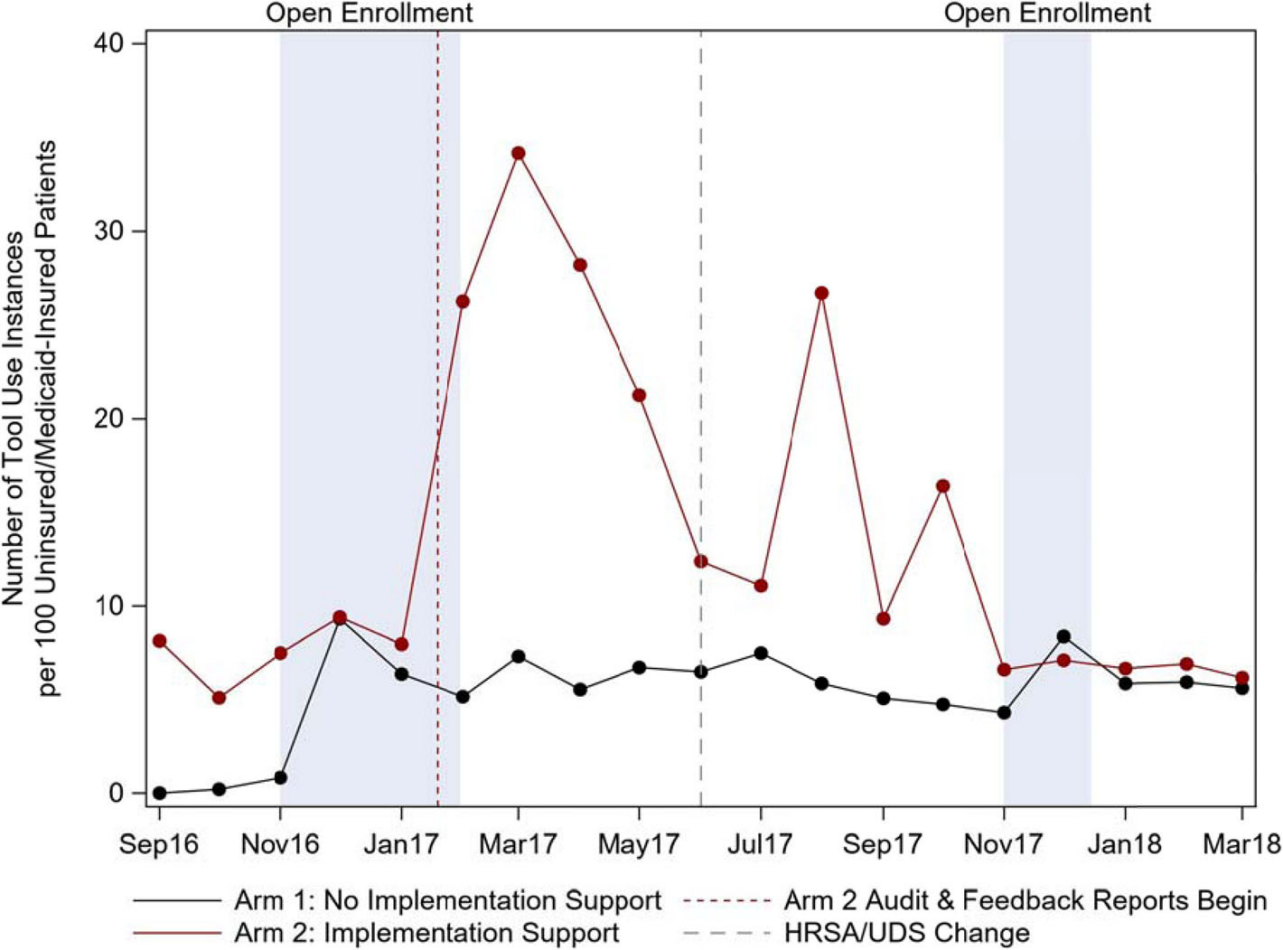
Rate of tool use per month between Arm 1 and Arm 2 clinics, among ‘high risk’ patients with at least 1 Medicaid-insured or uninsured visit during the study period, Sept 2016-Mar 2018. *Note: ‘HRSA (Health Resources and Services Agency) UDS (Unified Data Set) Change’ was a policy mandate that required health centers to report on health insurance enrollment assistance provided. This change was hypothesized to potentially impact tool utilization

The monthly number of tool use instances per 100 uninsured or Medicaid-insured patients varied over time for health centers in both study arms. Arm 2 CHCs had a high rate of tool use initially which peaked sharply in March of 2017, and subsequently decreased with multiple smaller surges, ending at a rate similar to Arm 1. Utilization in Arm 1 increased sharply in month 3 of the project and remained approximately steady afterward. (Fig. 1).

**Fig. 2:**
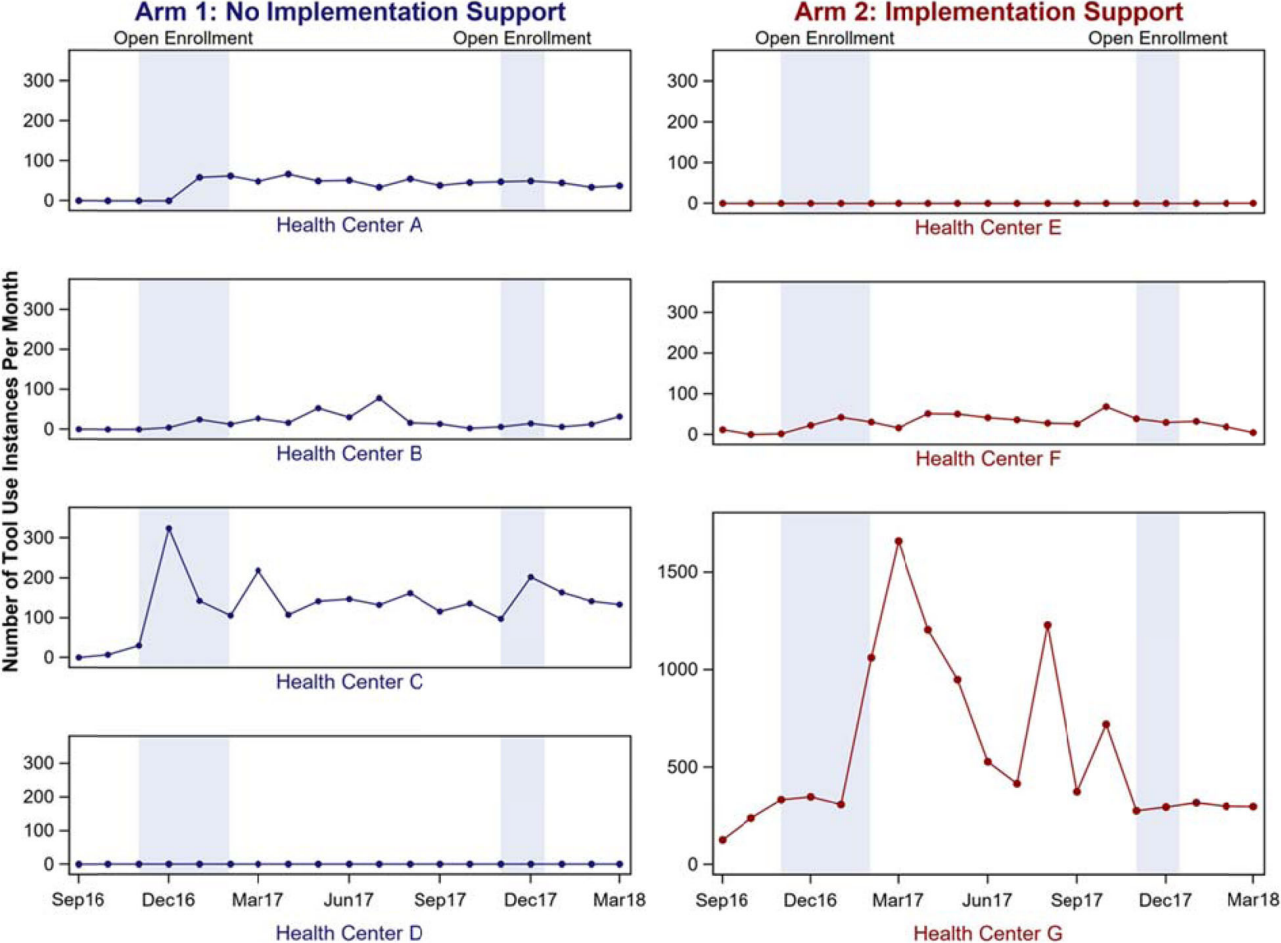
Total monthly instances of tool utilization by health center, September, 2016 – March, 2018

Stratifying the total instances of tool utilization by health center (Fig. 2) demonstrates that the shape of the Arm 2 line in Fig. 1 is similar to the shape of the line of tool utilization for Health Center G. In some health centers, small peaks of tool utilization were evident during open enrollment periods, most notably at Health Center C. Variability of total tool use over time was impacted by multiple factors including leadership engagement and implementation climate (described below).

### Perceived relative advantage played an important role in implementation

Three CFIR elements were identified as influencing use of the enrollment tool at the health centers: relative advantage, implementation climate, and leadership engagement. Table 5 provides a definition of these three CFIR elements and corresponding qualitative data examples to illustrate how health centers varied from low to high on each element. Table 6 shows the variation of the CFIR elements across health centers.

**Table 5:** CFIR Elements and implementation: qualitative examples

**Relative Advantage**	High	“You know, [the enrollment tool] sure beats the notes that you’d have to put in. I mean before it was, you know, note after note. Now there’s a place for a comment and there’s a place for what you did, and you just click different things. It’s really quick … I think you tend to capture more of the people you helped.” ***Enrollment assister Interview, Health Center A***
Stakeholders’ perception of the advantage of implementing the intervention versus an alternative solution	Low	“We talk to the assister about what she would like to see in the [enrollment] tool. She wants to have multiple boxes so that each family member, their DOB, and Medicaid number can be all the same form, and she would like a tool that would be good for tracking. Her supervisor asks what she likes better, the [enrollment] tool or the Access database system they used previously. The assister immediately and emphatically says that the Access database was better.” ***Fieldnotes, Health Center C***
**Implementation Climate**	Strong	“… Our EPIC clinic applications team really owned the training of the [enrollment] tool. So we sat down in a group [with assisters], and we had a guide … a step by step, here’s what you do. And then we logged into computers, all in the same room, and practiced with it as well …” ***Operations Manager Interview, Health Center G***
The absorptive capacity for change, shared receptivity of involved individuals to an intervention, and the extent to which use of that intervention will be rewarded, supported, and expected within their organization		
	Weak	“I don’t know if we got an email or what it was. The [EHR specialist] said that starting October 1st … we would have to use it so it made it sound like it was not an option, and I will be honest, we were not happy about making it, but we made the changes and so we did start using it as of August 1st. We did have a lot of hiccups in the beginning … I didn’t read the instructions as thoroughly as I should have, but it wasn’t well received in the beginning.” ***Enrollment assister Interview, Health Center C***
**Leadership engagement**	Strong	“Our goal, or hope as an FQHC is to provide care for every single Medicaid-covered person in the county... With the alternative payment model and with some of the, sort of incentives or quality metrics that [our Accountable Care Organization] has put in front of us, we definitely need to be doing more outreach. And I think that’s where the [enrollment tool] helps a lot … Yeah, it’s very helpful to be able to track that and-, and keep ahead of that because, um, the Medicaid system is our best payer.” ***Chief Financial Officer, Interview, Health Center F***
Commitment, involvement, and accountability of leaders and managers with the implementation.		
	Weak	“I [sighs] am very upfront and open about the fact that the outreach worker position is an area that I don’t know much about. It was put under me kind of as an afterthought. … Someday I would like to know more about all of that stuff, and what the tools look like and what the process is and where we can go from there. But right now, it’s just like – it’s the next thing on my agenda. ***Behavioral Health Director [and head of department that includes enrollment assisters] Interview, Health Center A***

**Table 6:** CFIR element ratings by health center

	Arm 1				Arm 2		
	**Health Center A**	**Health Center B**	**Health Center C**	H**ealth Center D**	**Health Center E**	**Health Center F**	**Health Center G**
Relative Advantage	High	Low / High^b^	Low	No data	Low	High	High
Implementation Climate	High	High	Low	No data	High	Low	High
Leadership Engagement	Low	High	High	None^a^	Low	High	High

^a^Leaders that agreed to implement the tool left the organization; new leaders were unengaged

^b^This practice did not see the advantage of this tool until team members discovered its HRSA reporting functionality. These additions changed practice members perceptions of relative advantage of using this tool from low to high

Health centers reported that the enrollment tool’s *relative advantage* over existing systems was one main factor driving tool utilization. The organizations with the most favorable views of the tool either had no previous system in place for tracking enrollment work, or systems they considered disorganized. The biggest advantages of the enrollment tool were its utility in tracking enrollment applications during the period between an application’s submission and acceptance, and its usefulness for federal HRSA reporting. For example, Health Center E did not use the enrollment tool and instead used a different insurance tracking tool outside of their EHR system that better suited the needs of their staff (who did not always have access to the EHR). Health Center B started using the tool more after discovering its advantage of fulfilling HRSA reporting. Health Center G found the tool most beneficial when paired with Medicaid date of coverage data (the specific date on which a patient’s Medicaid insurance would expire) which they were intermittently able to acquire from a state-level partner and use for proactive patient outreach.

Some health centers reported that *implementation climate* also influenced tool utilization. Health centers with high implementation support, usually from technical staff or leadership, found that this support promoted consistency in tool use across assister teams; these health centers commonly used audit and feedback mechanisms to address issues and inconsistencies in enrollment tool use as they arose. This support also helped assister teams initially understand where tool use might best fit within their workflow, and how it might benefit their work. For example, a collaborative climate of partnership with the local Accountable Care Organization to receive health insurance coverage dates among patients in Health Center G occurred at the same time as the dramatic increase in tool utilization at Health Center G in early 2017. When this partnership was not sustained, there was a subsequent decrease in tool utilization. Health centers with a less favorable implementation climate, such as Health Center C, had assister staff members who found the tool to be burdensome, and some reported to be doubling up their work.

Finally, *leadership engagement* impacted how the tool was initially received at a health center and could drive tool use. Engaged upper and middle management leaders were able to facilitate buy-in for the tool from assister teams, especially when leaders conveyed excitement regarding how the tool might benefit their own work and/or conveyed the expectation to assisters that they use the tool, and that their work is part of a larger goal. This was exemplified in Health Center C which had a competing tool developed by one of the assisters, but the directive from leadership fostered use the enrollment tool. Conversely, unengaged health center leaders had little knowledge of the work that assisters do, nor knowledge of the enroll tool. In the case of Health Center D, leadership turned over and left no one at the health center engaged in tool implementation. As such, this health center did not use the tool at all.

## Discussion

We developed a novel, integrated EHR tool designed to support health insurance enrollment assisters at CHCs in performing O&E activities over time. During an 18-month implementation period, most health centers used the tool regardless of implementation strategy (basic educational materials in Arm 1 versus materials with facilitation in Arm 2). On average, CHCs that received facilitation (Arm 2) utilized the tool at a higher rate than did CHCs that received basic educational materials only (Arm 1). This observed difference between implementation groups is likely attributable to multiple factors including (a) a unique combination of perceived relative advantage, leadership engagement, and implementation climate that allowed a single health center (G) to drive much of the observed between-group differences, (b) the presence of facilitation, and (c) more immediate tool use among Arm 2 CHCs.

The value of individualized in-person facilitation has been demonstrated for a wide variety of clinic-based interventions [8, 22]. Notably, the facilitation provided in our project required substantially fewer resources than in other studies [23], which suggests that modest or low-intensity facilitation in the form of tailored regular outreach and stand-by support may be effective and important to consider for targeted primary care interventions. The value of this particular strategy of audit and feedback with individualized close follow up has been demonstrated elsewhere [24].

While health centers that received facilitation utilized the tool more on average across the study period, the fact that health centers without facilitation utilized the tool at a similar rate by the end of the study period suggests that basic educational materials might be sufficient for successful implementation, if given additional time. The more immediate tool utilization observed in Arm 2 may have been related to the presence of facilitation, engagement in beta testing prior to the study period, or due to intrinsic differences between health centers. Alternatively, with more intensive facilitation or additional support of outreach and enrollment activities outside of our enrollment tool, we might have observed a continued increase in utilization in Arm 2, rather than the observed plateau.

Qualitative interviews revealed that perceived relative advantage of the tool, above all else, drove decision-making regarding tool utilization within health centers. The most commonly reported relative advantage of the enrollment tool was the reporting functionality that assisted with generation of needed O&E tracking reports to satisfy HRSA grant requirements. Presence of this functionality was strongly motivating to health centers, and increased after these data became mandated metrics for the Uniform Data System (UDS) during the study period. Health Centers that found the tool to have a lower relative advantage suggested various improvements, including linkage of family members within the EHR, adding pregnancy status and other tracking functions, and flagging applications as urgent/non-urgent.

Barriers to tool uptake were also evident, particularly in Heath Centers D and E that chose not to engage with the tool. In health center D, dramatic staff turnover in leadership left minimal awareness of the tool’s presence. Health Center E utilized a purchased product (external to the EHR) to perform some of the same tracking functions as the enrollment tool and, though they initially planned to utilize the enrollment tool for a subpopulation, they ultimately decided not to use it. The complete lack of tool use from these two health centers suggests a need to more thoroughly assess clinic receptivity before investing in facilitated tool implementation.

Beyond the specifics of this study, as one of the first pragmatic hybrid implementation-effectiveness trials to be launched, our findings highlight two important and ongoing themes for future investigation in the emerging field of implementation science. (1) Implementation support may speed adoption, but the control group eventually saw similar results. When is this investment in implementation worth it? How might pre-implementation assessments be used to tailor support that maximizes its value? (2) An implementation plateau (or backslide to baseline) coincides with a “wind down” of the most active period of support. How do we develop and test more effective sustainability strategies? Is there a minimum level of readiness and receptivity in a given clinical setting before we invest in implementation support? (3) A complete lack of engagement from some clinics (one in each arm) suggests a need for additional engagement strategies to promote use-centered design. Future research is needed to identify what approaches might maximize this early participation.

### Limitations

Clinics volunteered to be part of the study; thus, they are not representative of the general population of CHCs. The overall participation rate of 32% was low. Reasons for non-participation in the project included staff turnover, competing priorities, already utilizing an alternative tool, and participation in alternative research/intervention projects. Thus, our subset of participating health centers may be more motivated, stable, and available than others. Despite a strong randomized design, the small number of participating health centers in each arm makes it difficult to generalize from the conclusions.

In this pragmatic trial, clinics in both arms decided how to implement the tool within their particular populations. This flexible implementation approach was a strength of the study, but it may have also created some disconnection between perceived and measured tool use which manifested as some discordance between qualitative themes and utilization rate (for example, low relative advantage and low implementation climate in a health center with high tool utilization). Similarly, a lower rate of tool utilization may represent less robust O&E activities overall, rather than incomplete implementation of the tool itself. Since there were no pre-specified external guidelines to health centers regarding how the tool ought to be used (for which populations and to what extent) it was not possible to assess whether health centers met expectations for tool use. This flexibility was necessary for the project because of the inherent differences in health center resources and structures, though it does complicate interpretation of the ultimate success of tool implementation.

The primary outcome of this study was the rate of tool utilization overall, but given that the enrollment tool had many layers of functionality (Appendix Table 1), it is possible that some differences in tool utilization strategy were not measured between groups. For example, health centers may differ in their utilization of tool functionalities with some using the tool only for HRSA reporting, and others using the tool more robustly to assist with proactive insurance outreach, as noted above with Health Center G. Since Arm 2 health centers experienced multiple components of support (beta testing and implementation support) as well as some situational benefits (such as the partnership between Health Center G and their local accountable care organization) we cannot assess the impacts of each of these components individually.

### Conclusions

Health centers are increasingly active in supporting patients and other community members in enrolling in health insurance. Our EHR-based tracking tool was adopted by some health centers. The tool implementation in this project suggests promising value of lower intensity facilitation which improved initial adoption and utilization, especially relevant to community health centers with limited resources. However, decline in utilization after facilitation period highlights the need to a better understanding of how much facilitation is needed and for how long to achieve sustainability.

## Supplementary information

**Additional file 1:Appendix Figure 1**. CONSORT diagram. **Appendix Table 1.** Interview guide for health insurance assistors. **Appendix Table 2.** Enrollment Tool Field Definitions for Documentation and Tracking. **Appendix Figure 2.** The enrollment tool.

## Data Availability

The datasets generated and/or analyzed during the current study are not publicly available in compliance with the Health Information Portability and Accountability Act but deidentified data could be made available from the corresponding author on reasonable request.

## Abbreviations

HIT: Health information technology
CHC: Community health center
ACA: Affordable Care Act
HRSA: Health Resources and Services Administration
O&E: Outreach and enrollment
EHR: Electronic health record
PBRN: Practice based research network
FQHC: Federally Qualified Health Center
FPL: Federal poverty level
CFIR: Consolidated framework for implementation research
UDS: Uniform Data System
