# Supplementary material for: Implementation and adoption of a health insurance support tool in the electronic health record: a mixed methods analysis within a randomized trial
Source: BMC Health Serv Res. 2020 May 15;20:428. doi: 10.1186/s12913-020-05317-z (PMC7227079; doi:10.1186/s12913-020-05317-z)

**APPENDIX:**

Appendix Figure 1. CONSORT diagram

Declined to participate (n=15)

Potentially eligible health centers (n=83)

Eligible for recruitment (n=32)

Invited to participate (n=22)

Not invited to participate due to active recruitment for another study (n=10)

Excluded (n=51)

- No EHR data prior to 1/1/13 (n=26)

- Left OCHIN network (n=3)

- Prior participation in a similar study (n=3)

- Medicaid non-expansion states (n=11)

- Health center does not deliver primary care (n=8)

Randomized (n=7)

Arm 2

Tool + basic educational materials + Practice Facilitation (n=3)

Arm 1

Tool + basic educational materials (n=4)

Appendix Table 1. Interview guide for health insurance assistors

| INTRODUCTION   - **Currently, could you describe what you do on an average work day?**   - What are your weekly patterns?   - What kind of monthly or annual responsibilities do you have? - **Can you describe your current advocacy work?**   - Why is this type of work important for the clinic?   - What are the specific challenges involved with serving homeless clients?   - To what extent to do expect this work to continue after you retire? - **How do you typically interact with [backup Assister]?**   - What kinds of tasks does she usually help you with?   ENROLLMENT AND RENEWAL PROCESSES  Could you describe the process of enrolling someone for insurance for the first time? It might help to imagine a recent client/patient and walk me through what you did step by step.   - **When does this vary? For whom?** - **What is difficult about this process for you?**   - How do you resolve this? - **What barriers do you see clients face?**   - How does this affect the process of getting them insured? - **What is the timeline for getting insured?**   - What about same day appointments? - **How do you document all these steps?**   - What are the most important pieces of information to document?     - Why?   - Who else in the organization needs to know this? - **What do you do differently for a renewal application?**   - Could you walk me through the process for renewing a client’s insurance?   MONITORING INSURANCE STATUS   - **How involved are you with assessing whether or not clients have insurance when they have an upcoming appointment?** - **Could you tell me step by step what you do to find out about a client’s insurance?**   - What do you do if the client does not have active insurance?   - Who else is involved with this process? - **How do you know if a client’s insurance is going to term in the near future?** - **How do you see your role helping keep patients insured?**   - What do you consider your responsibility regarding coverage continuity?   - How do you go about helping patients stay insured?   - What makes this challenging?   - What helps you do this? - **What level of responsibility do you think the clinic should have for keeping patients insured?**   TOOL IMPLEMENTATION   - **Can you tell me about how the enrollment tool was originally implemented in your practice?** - How did you hear about the tool? - Who were you communicating with about the tool? - **What was your understanding of why the enrollment tool was being implemented?** - What did you think about the change to a new tool? - What was your initial opinion of the tool? - Probe: irritation, more work, excitement, change fatigue, confusion   What did others in the practice think about the tool?   - **How did you go about training [backup Assister] to use the enrollment tool?**   - Probe: LMS materials, OCHIN video, toolkit, and in house documents used.   - What would helpful for training other new assisters in the clinic?   TOOL USE OVERVIEW   - **What do you see as the purpose of this tool?**   - How does it fit into [Health Center]’s work?     - Probe: Why did the organization want it? - **What are your goals with using the enrollment tool?** - **How well has the enrolment tool lived up to your expectations?**   - Could you give me an example? - **Could you describe the differences in what you do now that you use the enrollment tool to prior?** - Probe: Change in workflow, ease of communicating insurance status, outreach. - **How does the tool support your current workflows?**   - Where do you struggle with the tool?   - What would improve the enrollment tool for your work? - **How is the tool being used across the clinic, and across departments?**   - How often are providers using the tool? - **What advice would you give other practices about the tool?**   - For implementing the tool?   - For using the tool?   MOVING FORWARD   - **What are your overall hopes for [Health Center x]’s Outreach and Enrollment Team as you transition out of your role?** - **Can you describe [new Assister]’s onboarding process?**   - What types of trainings is she doing?     - How are you training [new Assister] to use the tool?   - To what extent will [new Assister] be involved in patient advocacy work? - **What else would you like to share about your experiences with this study and with the enrollment tool?** |
| --- |

Appendix Table 2. Enrollment Tool Field Definitions for Documentation and Tracking.

| Field Name | Description |
| --- | --- |
| **Status of Case** | **Category List:** Pending  Submitted  Closed  Information Only |
| **Application Pending Reason** | **Selection List:** Application sent home  Additional signature  Date of request  Date of birth  Immigration documents  Income  SSN |
| **Date of Request** | **Date Calendar**: Date of signature on the application |
| **Date to Follow Up** | **Date Calendar**: Date you want to follow up on communications with the patient or other information relating to the application. |
| **Notes** | **Free Text:** Use as needed. Best practice is to begin a brief note with today’s date for easy distinction as more notes are added on different dates. The text window expands as notes are added. |
| **Date Application Submitted** | **Date Calendar**: Date the application is submitted |
| **Medicaid Case Number** | **Alpha-Numeric:** Document if there is one. |
| **Name of Eligibility Specialist** | **Free Text:** Your name in a format consistent with your policy. (Example: Last name, first initial (no spaces) |
| **Date of Eligibility Specialist Meeting** | **Date Calendar**: Document as needed. |
| **Medical Insurance**  **Applied for** | **Selection List:** CA-Medicaid  OH-Medicaid  OR-Medicaid  Marketplace  Other Program |
| **Dental Insurance Applied For** | **Selection List:** CA-Medicaid  OH-Medicaid  OR-Medicaid  Marketplace  Other Program |
| **Consent Obtained?** | **Selection List**: No  Yes |
| **Barriers** | **Selection List:** Multiple barriers to the application process may be selected. |
| **Eligibility Status** | **Selection List:** Pending  Approved  Not Approved  Presumptive Eligibility |
| **Reason Not Approved** | **Selection List:** Immigration status  Income |
| **Insurance Effective Date** | **Date Calendar**: Date the insurance becomes effective |
| **Insurance ID** | **Alpha-Numeric:** Document when known |

| **Assists Provided** | | | Report the number of lives assisted per session |
| --- | --- | --- | --- |
|  | | Date | Date of assist |
|  | Number of Lives Assisted | | Number of individuals in a family being assisted at that session |
|  | | Re/New Assist | Check box |
|  | | Minutes | Time Eligibility Specialist assisted the patient |
|  | | Comments/Action | Free text section that can be used for notes and comments |
| **Applications Submitted** | | | Report the number of lives covered by the application |
|  | | Date | Date of assist |
|  | | Number of Lives | Number of individuals in a family being assisted at that session |
|  | | Medicaid | Type of Medicaid Health Plan applied for |
|  | | Marketplace (QHP) | Type of Marketplace Health Plan applied for |
|  | | Other |  |
|  | | Re/New | Check box |
|  | | Minutes | Time Eligibility Specialist assisted the patient |
|  | | Comments/Action | Free text section that can be used for notes and comments |
| **Estimated Enrolled** | | | Report the number of lives covered by the application |
|  | | Date | Date of assist |
|  | | Number of Lives | Number of individuals in a family being assisted at that session |
|  | | Medicaid | Type of Medicaid Health Plan applied for |
|  | | Marketplace (QHP) | Type of Marketplace Health Plan applied for |
|  | | Other |  |
|  | | Re/New | Check box |
|  | | Minutes | Time Eligibility Specialist assisted the patient |
|  | | Comments/Action | Free text section that can be used for notes and comments |

Appendix Figure 2. The enrollment tool


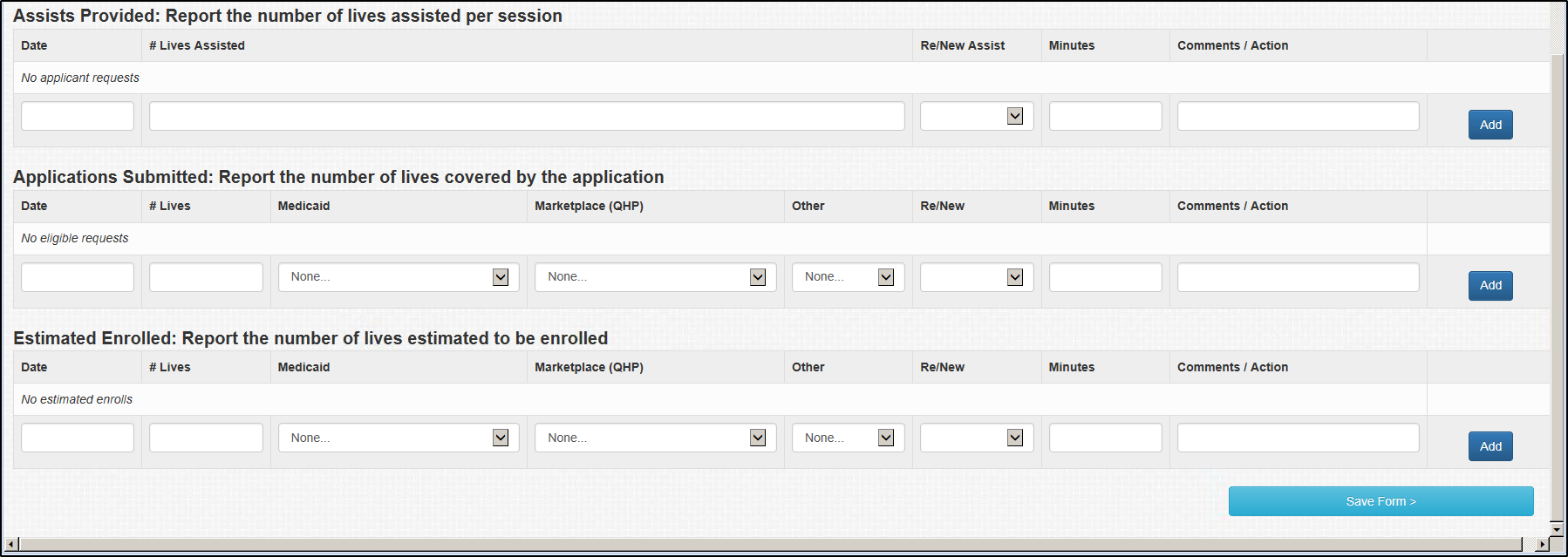

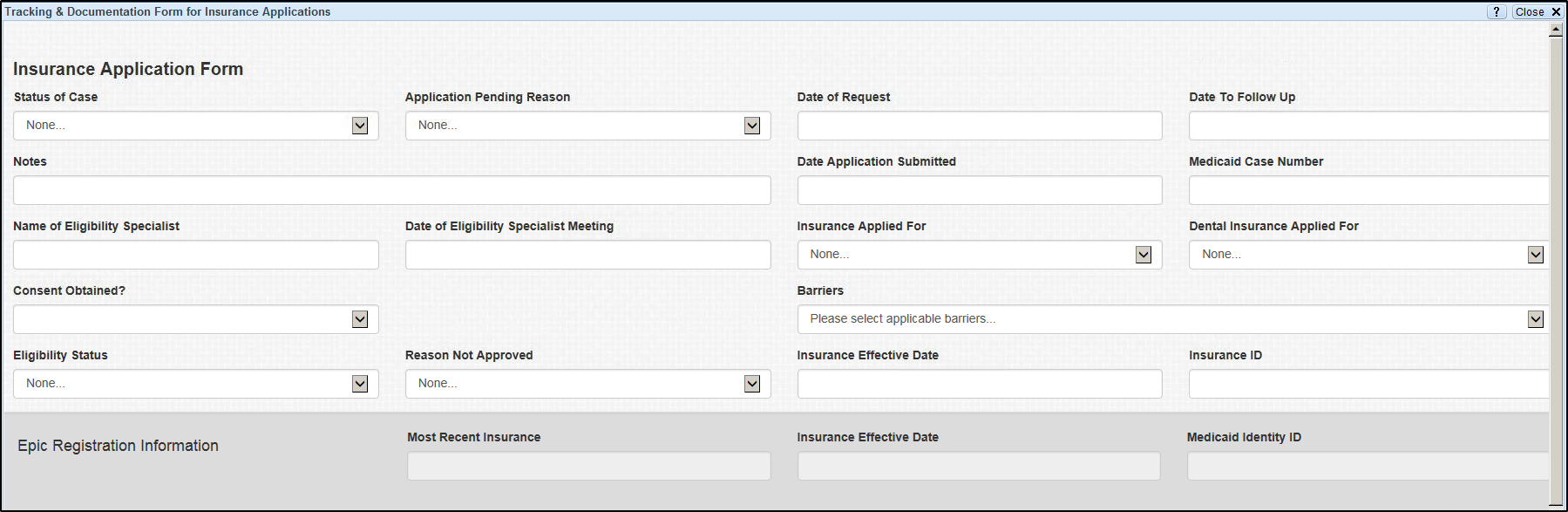

Supplement: Supplementary file 1 — Additional file 1:Appendix Figure 1. CONSORT diagram. Appendix Table 1. Interview guide for health insurance assistors. Appendix Table 2. Enrollment Tool Field Definitions for Documentation and Tracking. Appendix Figure 2. The enrollment tool. [file 12913_2020_5317_MOESM1_ESM.docx]
